# Is liver to lung shunting in colorectal liver metastasis the cause of toxicity following treatment with cytotoxic microsphere aggregates?

**DOI:** 10.1038/bjc.1992.429

**Published:** 1992-12

**Authors:** T. W. Hennigan, S. Earlam, T. G. Allen-Mersh

**Affiliations:** Department of Surgery, Charing Cross and Westminster Medical School, London, UK.

## Abstract

**Images:**


Br. J. Cancer (1992), 66, 1169–1170 © Macmillan Press Ltd., 1992

SHORT COMMUNICATION

### Is liver to lung shunting in colorectal liver metastasis the cause of toxicity following treatment with cytotoxic microsphere aggregates?

T.W. Hennigan, S. Earlam & T.G. Allen-Mersh

*Department of Surgery, Charing Cross and Westminster Medical School, Fulham Palace Road, London W6 8RF, UK.*

**Summary** Incorporation of cytotoxic drugs into microspheres reduces but does not eliminate systemic toxicity. The extent of liver to lung shunt was measured in 26 patients with colorectal liver metastasis. Liver to lung shunting correlated with proportion of liver replacement but did not exceed 4.4% and therefore is unlikely to cause systemic toxicity.

Microspheres embolise in the first capillary bed encountered and when given regionally into the hepatic artery do so in the liver (Kerr *et al.*, 1988). They may prolong tumour cytotoxic exposure by two mechanisms. Firstly, they maybe contain the cytotoxic agent and become trapped in the tumour and liver vessels with leaching out of the cytotoxic into tumour (Codde *et al.*, 1990). Secondly, they may be administered at the same time as the cytotoxic agent and thereby reduce drug washout from the tumour capillary bed by reducing tumour blood flow (Dahkil *et al.*, 1982; Gyves *et al.*, 1983).

Regional drug delivery using microspheres is also associated with a reduction in systemic toxicity compared with systemic delivery of cytotoxic agent alone (McArdle *et al.*, 1988). However, systemic toxicity including myelosuppression still occurs (Codde *et al.*, 1990; Anderson *et al.*, 1989; Goldberg *et al.*, 1990; Pfiefle *et al.*, 1985). This may be due to shunting of the microspheres through tumour-associated arteriovenous communications of greater diameter than the microspheres.

We have investigated the extent of this shunt in patients with colorectal liver metastases and determined whether it is related to extent of tumour liver replacement.

### Methods

### *Patients studied*

Twenty-six patients with colorectal liver metastases were studied prior to treatment by hepatic artery infusion chemotherapy using 5-fluorodeoxyuridine via a totally implantable pump (Infusaid, Norwalk, Massachusetts).

### *Proportion liver volume replaced by tumour*

An abdominal CT scan was performed within 3 weeks of the study. All the CT scan slices (1 cm thick) in which the liver appeared were studied. The outline of the liver and of intrahepatic metastases on each slice were traced on transparent paper. The transparency was projected onto a point grid. The number of points lying within each metastasis and within normal liver was counted. Liver and tumour point counts for each slice were summed to give total liver area replacement. This area replacement ratio is, by the Principle of Delesse (Delesse, 1847; Grunwald & Wicher, 1985), equivalent to ratio of volume replacement.

Following the CT scan an implantable infusion pump was inserted with the infusion cannula placed in the gastroduodenal artery.

### *Proportion of liver to lung shunting*

Within 2 weeks of pump insertion, 5 mCi of technetium 99 m labelled macroaggregated albumin stable for 6 h (20–40 μm diameter, Pulmolyte, Du Pont, Stevenage) was injected via the infusion pump side port directly into the gastroduodenal artery.

A gamma camera scan (Scintronix gamma camera, 1 min statis images) was performed and planar images obtained 2 min after $^{99}$Tc macroaggregated albumin administration and a background correction made. Two regions of interest were drawn on the images. The first around all of the liver (Figure 2) and the second around both lungs (Figure 1). Total lung counts were divided by the sum of liver counts to derive a proportion of counts found in the lung compared with the lung and liver counts.

### Results

The median proportion of the liver area replaced (and therefore, by the principle of Delesse, volume) was 19.5% (interquartile range = 6 to 27%). The median proportion of

**Figure 1** The bold line indicates the regions of interest encompassing the lungs with the liver visible below. In no case did the ratio of lung:liver counts exceed 4.4%.

Correspondence: T.W. Hennigan, 125 Balfour Road, London W13 9TW, UK.
Received and accepted 1 June 1992.

**Figure 2** The bold line indicates the region of interest encompassing the liver with the infusion pump port visible at the bottom left of the image.

liver to lung shunting was 1.3% (interquartile range 0.4% to 2.86%) and did not exceed 4.4%.

There was a weak but significant positive correlation ($r = 0.433$, $P = 0.027$) between the proportion of liver to lung shunt and the proportion of liver volume replaced.

### Discussion

This study confirms that liver to lung shunting of microspheres of a similar size to those used in treatment does occur in human colorectal liver metastases. However, the extent of the shunt was small and was always below 4.4% of the counts retained in the tumour and liver despite extensive liver replacement by tumour.

There was a correlation between the extent of the shunt and the proportion of liver replacement by tumour. This suggests that the shunt was associated with tumour. It may be that the shunt was via vessels greater than 20 $\mu$m diameter situated in and around the tumour. Alternatively, the tumour could affect the normal liver vasculature by increasing the diameter of the normal liver vessels to allow the passage of the microaggregates.

Since the extent of the shunting which was identified was small, it is unlikely that this could be the reason for the toxicity associated with microsphere treatment. Therefore, attempts to diminish systemic toxicity by using larger microspheres which would not pass through the shunt is unlikely to be effective in reducing toxicity. Toxicity with cytotoxic-containing microspheres is more likely to be due to the release of cytotoxic drug from the microspheres into the systemic circulation after administration.

S.E. is supported by Cancer Relief. We are grateful for the assistance of K. Jeyasingh of the Department of Nuclear Medicine for performing the gamma camera scans.

### References

ANDERSON, M., ARONSEN, K.F., BALCH, C., DOMELLOF, L., EKSBORG, S., HAFSTROM, L.O., HOWELL, S.B., KARENSON, R., MIDLANDER, J. & TEDER, H. (1989). Pharmacokinetics of intra-arterial mitomycin C with or without degradable starch microspheres (DSM) in the treatment of non-resectable liver cancer. *Acta Oncol.*, **28**, 219-222.

CODDE, J.P., BURTON, M.A., KELEHER, A.S.G. & GRAY, B.N. (1990). Reduced toxicity of adriamycin by incorporation into ion exchange microspheres: a therapeutic study using a rat liver tumour model. *Anticancer Res.*, **10**, 1715-1718.

DAKHIL, S., ENSMINGER, W.D., CHO, K., NEIDERHUBER, J., DOAN, K. & WHEELER, R. (1982). Improved regional selectivity of hepatic arterial BCNU with degradable microspheres. *Cancer*, **50**, 631-635.

DELESSE, A. (1847). Procede mechanique pour determines la composition des roches (extrait). *C.R. Acad. Sci. (Paris)*, **25**, 544-547.

GOLDBERG, J.A., KERR, D.J., WILMOTT, N., MCKILLOP, J.H. & MCARDLE, C.S. (1990). Regional chemotherapy for colorectal liver metastasis: a phase II evaluation of targeted hepatic arterial 5-fluorouracil for colorectal liver metastases. *Br. J. Surg.*, **77**, 1238-1240.

GRUNWALD, J. & WICHER, W. (1985). Ultrastructural morphometry of cultivated smooth muscle cells from normotensive and hypertensive rats. *Exp. Pathol.*, **27**, 91-98.

GYVES, J.W., ENSMINGER, W.D., VANHARKEN, D., NEIDERHUBER, J.E., STETSON, P. & WALKER, S. (1983). Improved regional selectivity of hepatic arterial mitomycin by starch microspheres. *Clin. Pharmacol. Ther.*, **34**, 259-265.

KERR, D.J., WILMOTT, N., LEWI, H. & MCARDLE, C.S. (1988). The pharmacokinetics and distribution of Adriamycin-loaded albumin microsphere following intra-arterial administration. *Cancer*, **62**, 878-882.

MCARDLE, C.S., LEWI, H., HANSEL, D.J., KERR, D.J., MCKILLOP, J. & WILLMOT, N. (1988). Cytotoxic-loaded albumin microspheres: a novel approach to regional chemotherapy. *Br. J. Surg.*, **75**, 132-134.

PFEIFLE, C.E., HOWELL, S.B. & BOCKSTEIN, J.J. (1985). Pilot study of intra-arterial floxuridine, mitomycin and doxorubicin in combination with degradable starch microspheres to treat primary amd metastatic tumours of the liver. *Cancer Drug Deliv.*, **2**, 305-311.


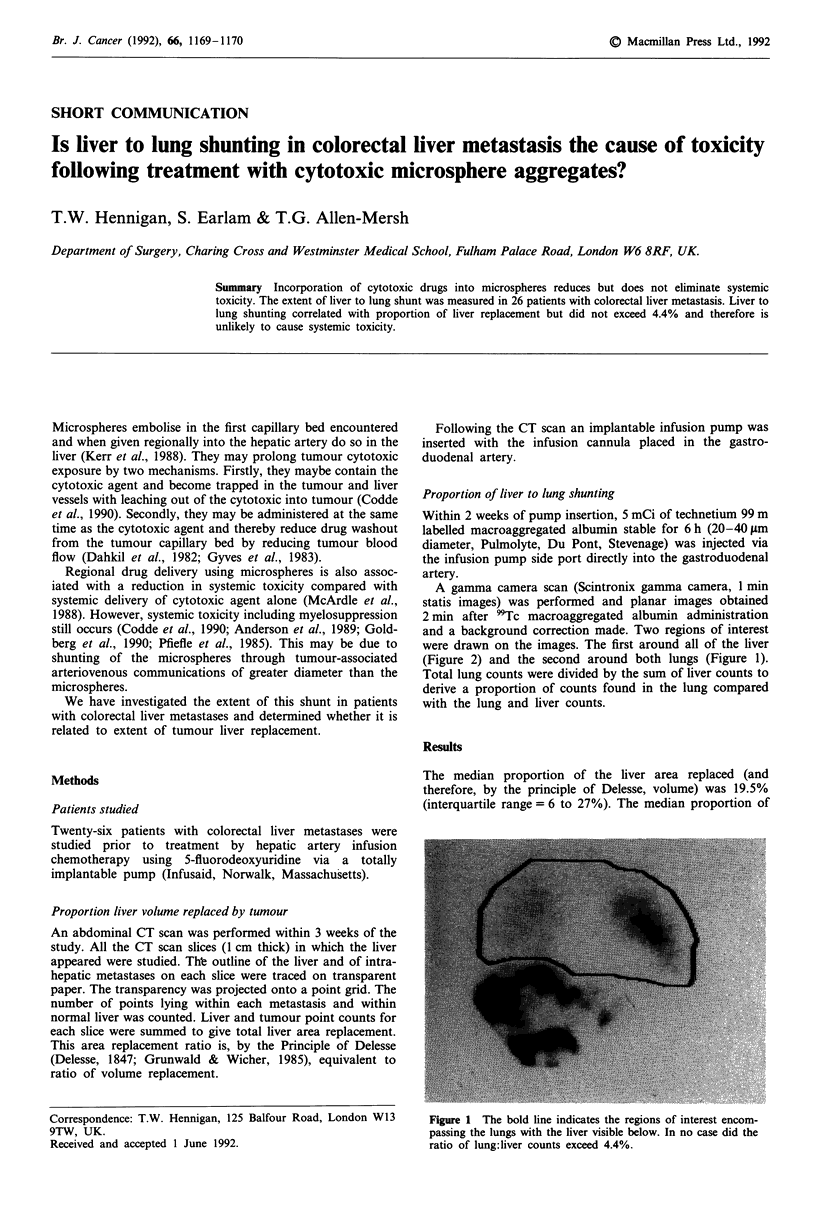

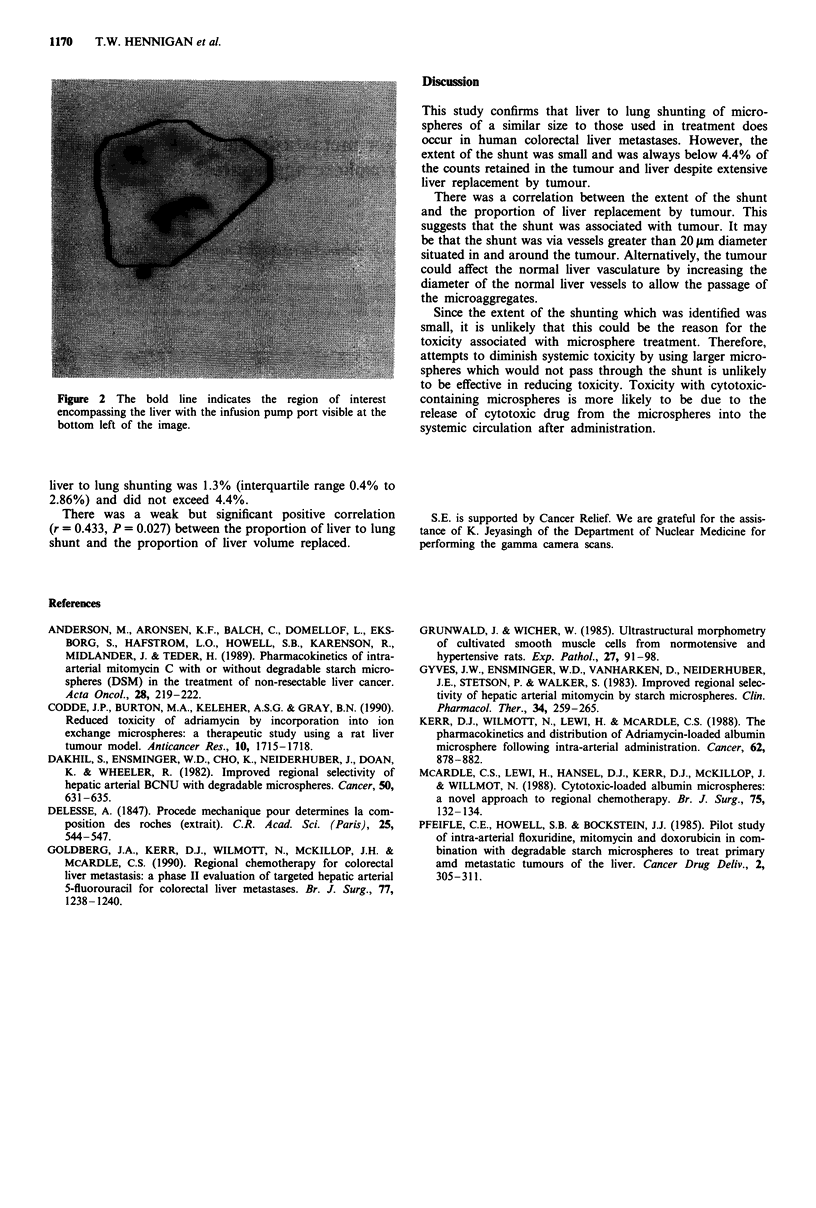


## References

[OCR_00156] Andersson M., Aronsen K. F., Balch C., Domellöf L., Eksborg S., Hafström L. O., Howell S. B., Kåresen R., Midander J., Teder H. (1989). Pharmacokinetics of intra-arterial mitomycin C with or without degradable starch microspheres (DSM) in the treatment of non-resectable liver cancer.. Acta Oncol.

[OCR_00162] Codde J. P., Burton M. A., Kelleher D. K., Archer S. G., Gray B. N. (1990). Reduced toxicity of adriamycin by incorporation into ion exchange microspheres: a therapeutic study using a rat liver tumour model.. Anticancer Res.

[OCR_00168] Dakhil S., Ensminger W., Cho K., Niederhuber J., Doan K., Wheeler R. (1982). Improved regional selectivity of hepatic arterial BCNU with degradable microspheres.. Cancer.

[OCR_00179] Goldberg J. A., Kerr D. J., Wilmott N., McKillop J. H., McArdle C. S. (1990). Regional chemotherapy for colorectal liver metastases: a phase II evaluation of targeted hepatic arterial 5-fluorouracil for colorectal liver metastases.. Br J Surg.

[OCR_00186] Grünwald J., Wischer W. (1985). Ultrastructural morphometry of cultivated smooth muscle cells from normotensive and hypertensive rats.. Exp Pathol.

[OCR_00191] Gyves J. W., Ensminger W. D., VanHarken D., Niederhuber J., Stetson P., Walker S. (1983). Improved regional selectivity of hepatic arterial mitomycin by starch microspheres.. Clin Pharmacol Ther.

[OCR_00197] Kerr D. J., Willmott N., McKillop J. H., Cummings J., Lewi H. J., McArdle C. S. (1988). Target organ disposition and plasma pharmacokinetics of doxorubicin incorporated into albumin microspheres after intrarenal arterial administration.. Cancer.

[OCR_00203] McArdle C. S., Lewi H., Hansell D., Kerr D. J., McKillop J., Willmott N. (1988). Cytotoxic-loaded albumin microspheres: a novel approach to regional chemotherapy.. Br J Surg.

[OCR_00209] Pfeifle C. E., Howell S. B., Bookstein J. J. (1985). Pilot study of intra-arterial floxuridine, mitomycin and doxorubicin in combination with degradable starch microspheres to treat primary and metastatic tumors of the liver.. Cancer Drug Deliv.

